# Congenital hallux valgus occurs in Fibrodysplasia Ossificans Progressiva and *BMPR1B-*associated dysplasia: an important distinction

**DOI:** 10.1186/s12920-024-01931-6

**Published:** 2024-06-15

**Authors:** Diksha Shirodkar, Sarah Francesca Smithson, Richard Keen, Tracy Lester, Benito Banos-Pinero, Christine Pamela Burren

**Affiliations:** 1 Department of Paediatric Endocrinology and Diabetes, Bristol Royal Hospital for Children, University Hospitals Bristol and Weston NHS Foundation Trust, Maudlin Street, Bristol, BS2 8BJ UK; 2 Department of Clinical Genetics, St Michael’s Hospital, University Hospitals Bristol and Weston NHS Foundation Trust, Southwell Street, Bristol, BS2 8EG UK; 3 Royal National Orthopaedic Hospital, Brockley Hill, Stanmore, Middlesex HA7 4LP UK; 4 Oxford University Hospitals NHS Foundation Trust, John Radcliffe Hospital, Headley Way, Headington, Oxford, Oxfordshire OX3 9DU UK; 5 Oxford Medical Genetics Laboratories, Oxford University Hospitals NHS Foundation Trust, The Churchill Hospital, Oxford, OX3 7LE UK

**Keywords:** BMPR1B mutations, Fibro-dysplastic Osseous dysplasia, Congenital bilateral hallux valgus

## Abstract

**Background:**

Fibrodysplasia Ossificans Progressiva (FOP; OMIM #135100) is an ultrarare genetic disorder characterised by congenital bilateral hallux valgus (CBHV), intermittent soft tissue swellings and progressive heterotopic ossification. We report a three-month-old girl with great toe abnormalities similar to FOP, in whom comprehensive clinical workup and genetic investigations illustrates an alternative diagnosis.

**Case presentation:**

A three-month-old girl presented with CBHV. The antenatal period was unremarkable, she was born by spontaneous vaginal delivery with an uneventful subsequent course, except for maternal concern of her bent toes which received reassurance from several health professionals. Her mother’s persisting concerns were explored via the internet and social media leading her to request referral to an expert bone centre for consideration of FOP. On examination, she was thriving, there was no dysmorphism, subcutaneous lumps, skeletal or extra-skeletal deformity except for shortened great toes with lateral deviation of the proximal and distal phalanges. FOP was a feasible diagnosis, for which CBHV is highlighted as an early sign. A cautionary potential diagnosis of FOP was counselled, including advice to defer intramuscular immunisations until genetic results available. Genetic investigation was undertaken through rapid whole genomic sequencing (WGS), with analysis of data from a skeletal dysplasia gene panel, which demonstrated no *ACVR1*variants. The only finding was a heterozygous variant of unknown significance in *BMPR1B* (c1460T>A, p.(Val487Asp)), which encodes a bone morphogenic receptor involved in brachydactyly syndromes A1, A2 and D and acromesomelic dysplasia 3 (only the latter being an autosomal recessive condition).

**Conclusion:**

This report highlights that CBHV serves as a vital diagnostic indicator of FOP and affected infants should be considered and investigated for FOP, including precautionary management whilst awaiting genetic studies. The second educational aspect is that CBHV may not represent a generalised skeletal disorder, or one much less significant than FOP. Receptor-ligand BMP and Activins mediated interactions are instrumental in the intricate embryology of the great toe. Recognition of non-FOP conditions caused by alterations in different genes are likely to increase with new genomic technology and large gene panels, enhancing understanding of bone signaling pathways.

## Background

The hallux valgus deformity usually occurs in adults and adolescents, whereas congenital bilateral hallux valgus (CBHV) is a rare entity which occurs primarily due to pre-existence of metatarsus primus varus resulting in compensatory distal angulation of the great toe [1, 2]. The most common cause (>98 %) is Fibrodysplasia Ossificans Progressiva (FOP), a condition of extra skeletal bone formation caused by gain-of-function variants in the *ACVR1* gene, which encodes a bone morphogenetic protein (BMP) type 1 receptor (Activin A Receptor Type 1) [3, 4]. Here we report the case of a three-month-old girl who was suspected of having this disabling condition on the basis of her bilateral great toe abnormality identical to that seen in children with FOP, in whom the genetic results revealed an unexpected finding denoting a less serious condition.

## Case presentation

**Fig. 1:**
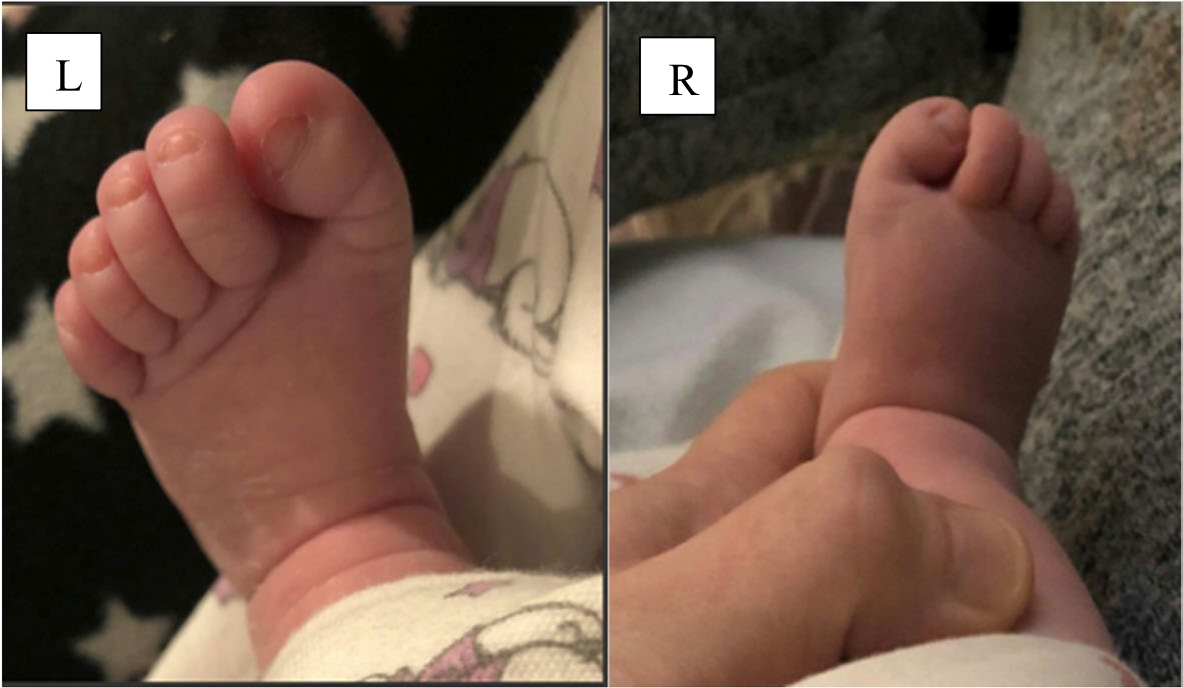
Clinical photographs demonstrating Bilateral hallux valgus (left [L] and right [R])

**Fig. 2:**
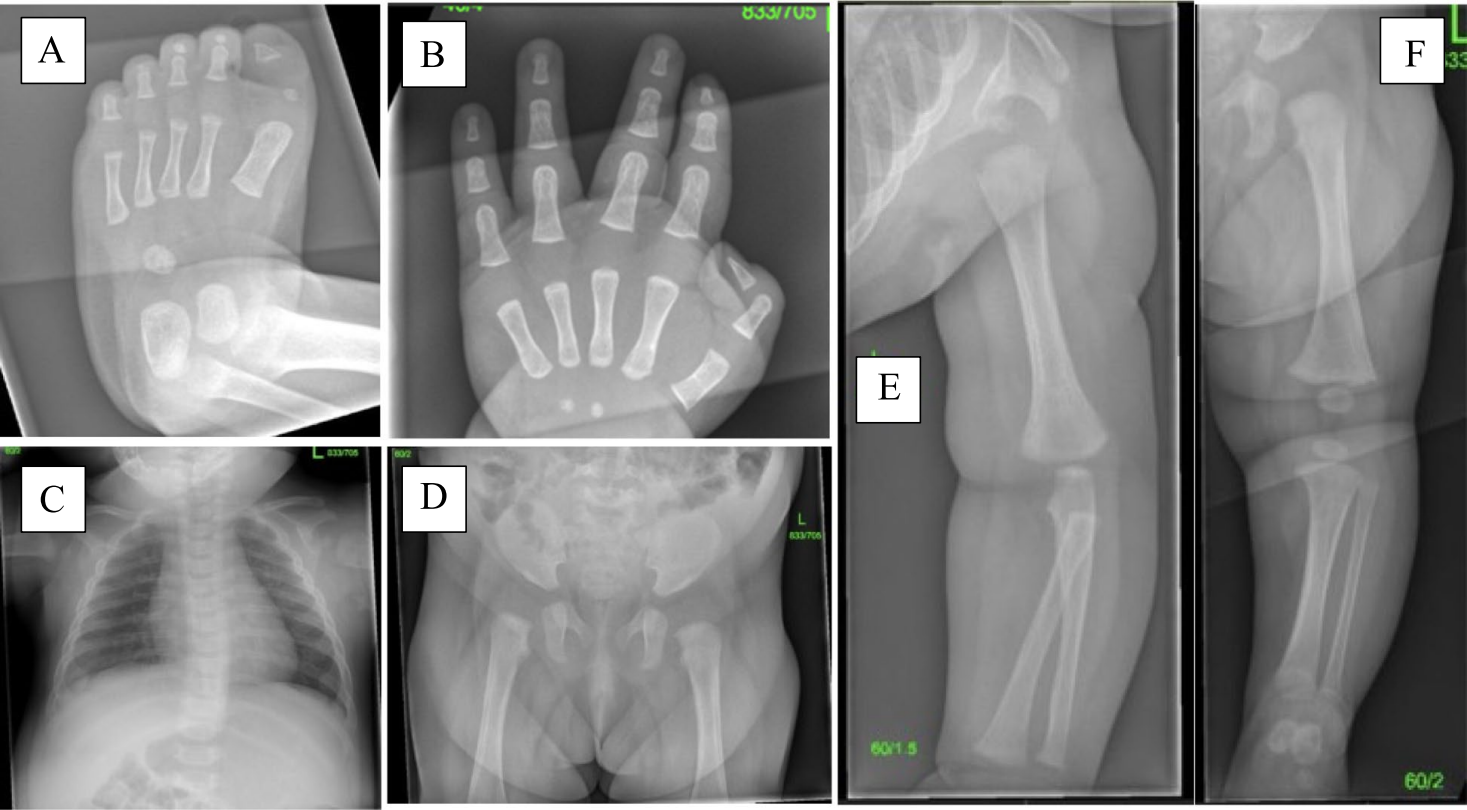
Skeletal survey plain radiographs. No areas of soft tissue ossification seen. **A**: Left foot demonstrating hallux valgus; a small squared proximal phalanx with a tapered distal phalanx with lateral deviation. **B**: The left hand no abnormalities identified. **C**: The chest illustrating a minimal thoracolumbar spine curvature was attributed to a positional effect and not scoliosis. **D**, **E** and **F**: Illustrate the pelvis with both hip joints, left shoulder joint with the arm and the left hip joint with the lower limb respectively demonstrating incidental physiological periosteal reaction of the humerus and femur

A three-month-old baby girl presented with CBHV. She was the only child of non-consanguineous parents, born by vaginal delivery with no antenatal or postnatal concerns and birth weight 2.95 kg. There was no family history of skeletal abnormalities. Her mother was concerned by the toe shape from birth. She was referred to our expert bone centre to explore the possibility of FOP. On examination the baby was well, thriving, weight 6.48 kg (75^th^-91^st^ centile), length: 59 cm (50^th^ centile) and head circumference 39 cm (50^th^ centile) with bilateral hallux valgus (Fig. 1). There was no facial dysmorphism, subcutaneous lumps, nor bony deformities elsewhere in the lower limbs, upper limbs, spine, or torso. The baby had a full range of movement at all joints of the trunk and limbs, except reduced flexion-extension of the interphalangeal joint of both great toes. The musculoskeletal examination was otherwise normal, including the absence of any ligamentous laxity. Biochemical bone profile was unremarkable. A skeletal survey confirmed bilateral hallux valgus, small square shaped proximal phalanx of the great toes, tapered distal phalanges with lateral deviation and no evidence of soft tissue heterotopic ossification (Fig. 2). The antero-posterior skull x-ray was reported to be normal. Minimal thoracolumbar spine curvature was attributed to a positional effect and not scoliosis. Incidental physiological periosteal reaction in the humeral and femoral shafts were also noted.

The new Genomic Testing Directory for England provides testing of *ACVR1* only within a large gene panel rather than via single gene sequencing. WGS libraries were prepared from blood DNA and sequenced commercially by Illumina. Data was processed via the Genomics England Rare Disease Pipeline v2.0, with reads aligned to reference build GRCh38 and variants called, using the DRAGEN software v3.2.22. Variants were then filtered using the virtual panel Skeletal dysplasia v2.2 (https://nhsgms-panelapp.genomicsengland.co.uk/). Detailed information on the variant prioritisation process and quality metrics used is in the Rare Disease Genome Analysis Guide v2.1 published by Genomics England (https://re-docs.genomicsengland.co.uk/rare_disease_2_2.pdf).

**Fig. 3:**
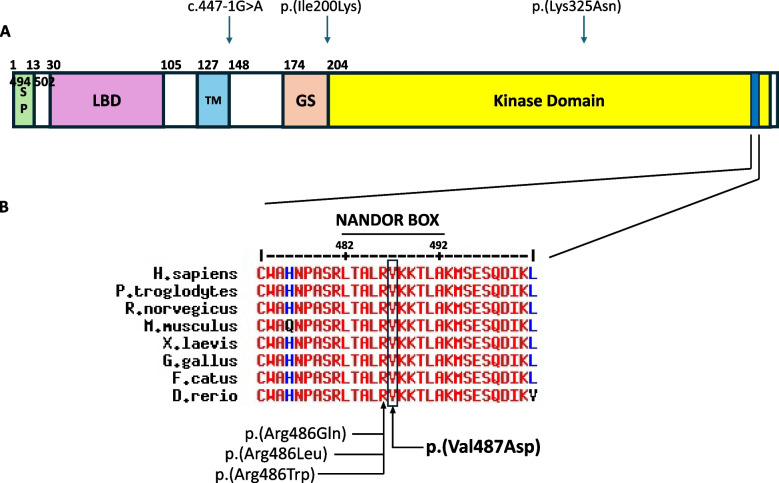
**A** Scheme of the BMPR1B protein showing domains. SP: signal peptide; LBD: Ligand-Binding domain; TM: transmembrane domain; GS: Glycine-Serine rich box. **B** Alignment of the NANDOR box of BMPR1B orthologues (https://multalin.toulouse.inra.fr/). The residue where our variant is located is marked in a black box with the variant labelled in bold black text. The location of other variants reported in the literature in association with autosomal dominant brachydactyly are indicated in schematics A and B in black text

Coverage of the *BMPR1A* gene was 100% at ≥15x calculated from reads with mapping quality >10 and >85x10^9 bases with Q≥30, after removing duplicate reads and overlapping bases after adaptor and quality trimming.WGS data analysed from the Skeletal Dysplasia Panel (R104 ~450 genes, including *ACVR1* which causes FOP) demonstrated a heterozygous variant of unknown significance (c.1460T>A, p.(Val487Asp)) in *BMPR1B* (Fig. 3). The variant was not recorded in the GnomAD database v4.1 [5]. Monoallelic pathogenic *BMPR1B* variants cause autosomal dominant brachydactyly (types A1, A2 and D) and biallelic pathogenic variants of *BMPR1B* cause acromesomelic dysplasia 3. Parental testing showed her mother had no *BMPR1B* gene changes and her father carries the same *BMPR1B* variant. Although the father has normal toe shape, he subsequently learnt that his predeceased mother, his older brother and his older brother’s three children had had the same abnormal toe shape as his daughter. These individuals were not available for genetic testing or detailed clinical examination, although on report the toe abnormality was an isolated clinical finding and not accompanied by additional musculoskeletal abnormalities, but this phenotypic history suggests this *BMPR1B* variant is of variable penetrance and in keeping with Autosomal Dominant Brachydactyly Type A.

## Discussion

Hallux valgus is a deformity commonly occurring in adults and adolescents [1]. The primary reason for this abnormality is the pre-existence of metatarsus primus varus leading to compensatory distal angulation of the great toe [1, 2]. Congenital and bilateral involvement is, however, rare. The causes for CBHV include deforming forces *in utero*, presence of an os intermetatarseum, an accessory bone between the first and second metatarsals, or presence of a supernumerary digit [3]. CBHV with absence or fusion interphalangeal joint (IPJ) is seen in more than 98% of children with FOP [4].

Other conditions associated with this anomaly include syndromes with symphalangism synostosis and brachydactyly [6]. CBHV can also occur an isolated congenital defect. FOP was initially called “Stone Man syndrome”, the term coined in the 16th century by Dr Guy Patin [7, 8] who described that man would be converted to a stone because of the painful hard lumps on his body. He also called this condition Myositis ossificans progressiva as he demonstrated bone formation in the muscles. Later in the 20th century, Dr Victor A. McKusick proved that this condition was not limited to the muscles but also the connective tissue including ligaments, fascia, aponeurosis and tendons at the point of local injury and hence the term Fibrodyplasia Ossificans Progressiva (FOP) came into existence [9]. FOP is an ultrarare genetic disorder with an incidence of 1 in 2 million characterised by ectopic ossification of connective tissues and the skeletal system leading to fusion of axial and appendicular skeleton [10]. Progressive heterotopic ossification (extra-osseous) presenting as palpable masses/lumps (100%) which are spontaneous or triggered by soft-tissue trauma (vaccinations/surgical procedures). The initial soft tissue swellings (flare-ups) are painful and precede the heterotopic ossification [11]. FOP can be diagnosed prenatally by ultrasound scans, if CBHV is present [12].

CBHV is the main presenting feature in infancy; less common features are scalp nodules/lumps and restricted limb reduction defects. Other clinical features include osteochondromas (proximal tibia), cervical spine fusion (mostly C2-C7), broad femoral neck, scoliosis, thumb malformation and limb reduction defects (sometimes misdiagnosed as brachydactyly syndromes) [11]. Bony lumps develop though the mechanisms of mononuclear infiltrate aggregation, then fibroproliferative changes and ultimately mature bone formation in non-bone tissues [6]. There are no biochemical abnormalities [6].

Several websites accessible to the general public and health professionals are valuable in raising disease awareness; these include the International FOP Association (IFOPA) [13], Focus of FOP [10] and FOP Friends [14]. Ready access to these by the lay public is demonstrated by our case and prompted the family to seek referral. As well as general public awareness, health professional awareness of CBHV and FOP are essential for referral pathways to expert bone assessment to flow effectively. Recognition of the potential significance of the CBHV in newborns is crucial to secure early diagnosis of FOP before emergence of calcification to facilitate careful preventative management, such as inappropriate intramuscular immunisation. Interventional clinical research trials are underway.

Although there is a very high association of CBHV and FOP, the embryological development of the great toe has an intricate pathway. Bone morphogenetic proteins (BMPs) belong to the TNF‐β family and play important roles in morphogenesis, specifically during early development and functions of various organ systems [15, 16]. BMP signals are conducted through either canonical or non‐canonical pathways [17]. The BMP receptors (BMPRs) have been classified in two groups: type I, containing the activin receptor‐like kinases (ACVR1) and type II, containing three receptors: BMPR2, ACVR2A, and ACVR2B. The type I receptors are further subdivided into three groups: BMPR1, ACVR1 and TβR‐I group [18]. Aberrations in BMPR1 (BMPR‐IA and BMPR‐IB) receptors, coded by the same gene, have been associated with brachydactyly type A along with pulmonary arterial hypertension [19], and autosomal recessive acromesomelic dysplasia [20]. Imbalance between the receptor-ligand concentrations and inappropriate signalling via the BMP pathways lead to aberrant activation of the BMP pathway resulting in the malformation of the great toe, which is extremely sensitive to these developmental disturbances [21]. In the patient described in this report, there was a heterozygous *BMPR1B* gene variant of unknown significance(figure ; this gene encodes the BMP type I receptor, sited in the same pathway as *ACVR1*-induced BMP signalling.

Similar to our patient, Towler *et al* described a child with CBHV (without any other limb abnormality) with a large intragenic deletion in BMP receptor 1B gene (*BMPR1B*) highlighting that CBHV is not exclusive to the diagnosis of FOP (*ACVR1* mutation) and that the receptor-ligand BMP pathways affect toe development [22]. Similarly, case reports demonstrate that CBHV occurs in brachydactyly type 2A, brachydactyly type C/symphalangism and in patients with isolated *BMPR1B* variants. Homozygous pathogenic variants of *BMPR1B* can cause acromesomelic dysplasia with malformations in the genital tract [20, 23, 24]. The identification of a BMPR1B variant (c.1460T>A p.(Val487Asp)) prompts the clinician to consider potential alternative skeletal dysplasias. These were discounted in this case in the absence of accompanying clinical or radiological features supportive of syndromes such as Brachydactyly, type A1, D and A2 nor of skeletal dysplasias such as acromesomelic dysplasia. A detailed outline of each of those is beyond the scope of this particular report. The p.Val487 residue is highly conserved across species and in 5/12 paralogues, and is located within nonactivating-non-downregulating (NANDOR) box within the kinase domain of the BMPR1A protein. Other variants within the NANDOR box, which has been proposed to have a role within receptor endocytosis and inactivation by transphosphorylation ([25–28http://www.uniprot.org/]) have been reported in association with brachydactyly ([25–27http://www.uniprot.org/]), see Fig. 3. Multiple in silico tools predict the p.(Val487Asp) variant to have a deleterious effect (REVEL score 0.958) [29]. CBHV remains an important and early presenting cardinal feature of FOP, but mechanistically reflects aberrant activity in the BMP pathways of the great toe formation, which can occur in several clinical conditions. In our case, although we cannot confirm the significance of the *BMPR1B* variant of uncertain significance, this phenotypic history of five additional affected members in the paternal family (not available for genetic testing) suggests a *BMPR1B* variant of variable penetrance and in keeping with Autosomal Dominant Brachydactyly Type A.Isolated CBHV itself is a rare skeletal deformity and to attribute its cause to a non-FOP gene variant without any other skeletal deformity contributes to the uniqueness of this case report. It illustrates that making a clinical diagnosis of FOP can be made provisionally, emphasising the importance of confirmatory genetic results. The implementation of new genomic technologies utilising panel testing in place of single gene testing may well identify more individuals with non-FOP gene variants within the *ACVR1*/BMP pathways.

## Conclusion

This case report highlights the importance of considering a range of causes of CBHV. Consideration of FOP remains the most crucial and urgent provisional diagnosis and this case illustrated the beneficial role of lay access to medical information on the internet in shortening the diagnostic journey in rare bone disease. Whilst awaiting genetic results we need to counsel with caution around FOP and delay intramuscular vaccinations until genetic confirmation is received. This case report also teaches us the importance of improving awareness around the condition, consider relevant differential diagnoses and benefits and cautions of whole genome sequencing rather than single gene testing.

## Data Availability

The datasets used and/or analysed during the current study available from the corresponding author on reasonable request.
